# Feature Selection Methods for Robust Decoding of Finger Movements in a Non-human Primate

**DOI:** 10.3389/fnins.2018.00022

**Published:** 2018-02-06

**Authors:** Subash Padmanaban, Justin Baker, Bradley Greger

**Affiliations:** ^1^School of Biological and Health Systems Engineering, Arizona State University, Tempe, AZ, United States; ^2^Viscus Biologics, Cleveland, OH, United States

**Keywords:** feature selection, neural decoding, principal component analysis, non-human primate, support vector machine

## Abstract

**Objective:** The performance of machine learning algorithms used for neural decoding of dexterous tasks may be impeded due to problems arising when dealing with high-dimensional data. The objective of feature selection algorithms is to choose a near-optimal subset of features from the original feature space to improve the performance of the decoding algorithm. The aim of our study was to compare the effects of four feature selection techniques, Wilcoxon signed-rank test, Relative Importance, Principal Component Analysis (PCA), and Mutual Information Maximization on SVM classification performance for a dexterous decoding task.

**Approach:** A nonhuman primate (NHP) was trained to perform small coordinated movements—similar to typing. An array of microelectrodes was implanted in the hand area of the motor cortex of the NHP and used to record action potentials (AP) during finger movements. A Support Vector Machine (SVM) was used to classify which finger movement the NHP was making based upon AP firing rates. We used the SVM classification to examine the functional parameters of (i) robustness to simulated failure and (ii) longevity of classification. We also compared the effect of using isolated-neuron and multi-unit firing rates as the feature vector supplied to the SVM.

**Main results:** The average decoding accuracy for multi-unit features and single-unit features using Mutual Information Maximization (MIM) across 47 sessions was 96.74 ± 3.5% and 97.65 ± 3.36% respectively. The reduction in decoding accuracy between using 100% of the features and 10% of features based on MIM was 45.56% (from 93.7 to 51.09%) and 4.75% (from 95.32 to 90.79%) for multi-unit and single-unit features respectively. MIM had best performance compared to other feature selection methods.

**Significance:** These results suggest improved decoding performance can be achieved by using optimally selected features. The results based on clinically relevant performance metrics also suggest that the decoding algorithm can be made robust by using optimal features and feature selection algorithms. We believe that even a few percent increase in performance is important and improves the decoding accuracy of the machine learning algorithm potentially increasing the ease of use of a brain machine interface.

## Introduction

Microelectrode array brain machine interfaces (BMI) have shown the potential to alleviate various neurological disorders. BMIs utilizing advances in robotics and machine learning can restore limited lower and upper extremity motor function. Several research studies have investigated the viability of a cortical brain machine interface in humans and NHPs (Carmena et al., 2003; Shenoy et al., 2003; Musallam et al., 2004; Hochberg et al., 2006; Santhanam et al., 2006; Kim et al., 2008; Ganguly and Carmena, 2009; Kellis et al., 2010; Ethier et al., 2012; Gilja et al., 2012, 2015; Collinger et al., 2013; Hwang and Andersen, 2013; Aflalo et al., 2015; Little et al., 2017). BMI for controlling a robotic limb or moving a cursor have been successfully demonstrated in humans and non-human primates (NHP). These systems provided real time control of a neuroprosthetic system by decoding neural signals moment by moment with an objective to provide certain functionality to replace the native arm. These systems are based on decoding the endpoint goal of reach and map the neural signals to spatially distributed targets. Wang et al. (2009) decoded individual finger movements using neural data recorded using a customized micro-ECoG grid. The quality of neural data was analyzed by using frequency domain based characteristics like coherence between different electrodes, modulation of neural signals and accuracy of finger movement classification. Shenoy et al. (2007) developed a finger movement classification algorithm based on neural data recording using Electrocorticographic BCI. The classification error achieved using this real-time BCI was 23%. Kubánek et al. (2009) also demonstrated the ability to decode the time course of individual finger flexions based on ECoG signals recorded from the motor cortical region in human subjects. (Graimann et al., 2004) developed a wavelet packet analysis and genetic algorithm for detecting ERPs in a single channel ECoG brain computer interface. Bashashati et al. (2007) and Garrett et al. (2003) provide a comprehensive review of feature selection methods in EEG-based brain computer interfaces.

BMIs can be broadly classified based on the type of bio-signal used to control the prosthesis. Electroencephalogram (EEG), Local field potential (LFP), and Action potential (AP) constitute the majority of source signals used in BMI. APs are discrete spiking events of an individual neuron. In statistics terms, APs or neural “spiking” can be thought of as a non-stationary point process in which neural information is largely encoded by changes in the AP firing rate coding (frequency of APs/spiking) (Truccolo et al., 2005). In this paper, we utilize neural recordings of APs from individual neurons to classify various movements of the fingers. One of the important characteristics of the human upper extremity functioning is the ability to perform coordinated and dexterous finger movements. Typing, eating with a spoon, writing with a pen and opening a lock with a key are some of the examples in our daily life that require such dexterous manipulations using individual or combined finger movements. Incorporating dexterity as a feature in a neuroprosthesis would help amputees and paralyzed persons to carry out a wider range of tasks. To achieve such dexterous control requires a neural decoding algorithm that can map high-dimensional neural signals onto a high-dimensional hand prosthesis. Optimizing algorithms for decoding neural signals will be critical for providing useful control of upper extremity neuroprosthesis. Feature selection is an important step in designing a machine learning system. Choosing a *O*-dimensional subset from a *P*-dimensional feature space consisting of “*P*” predictors using an objective metric is the aim of feature selection. Feature selection also reduces the dimensionality of feature space, inundating it with more “informative” features thus, removing lesser contributing ones that might occlude the feature space.

### Curse of dimensionality

**Figure 1:**
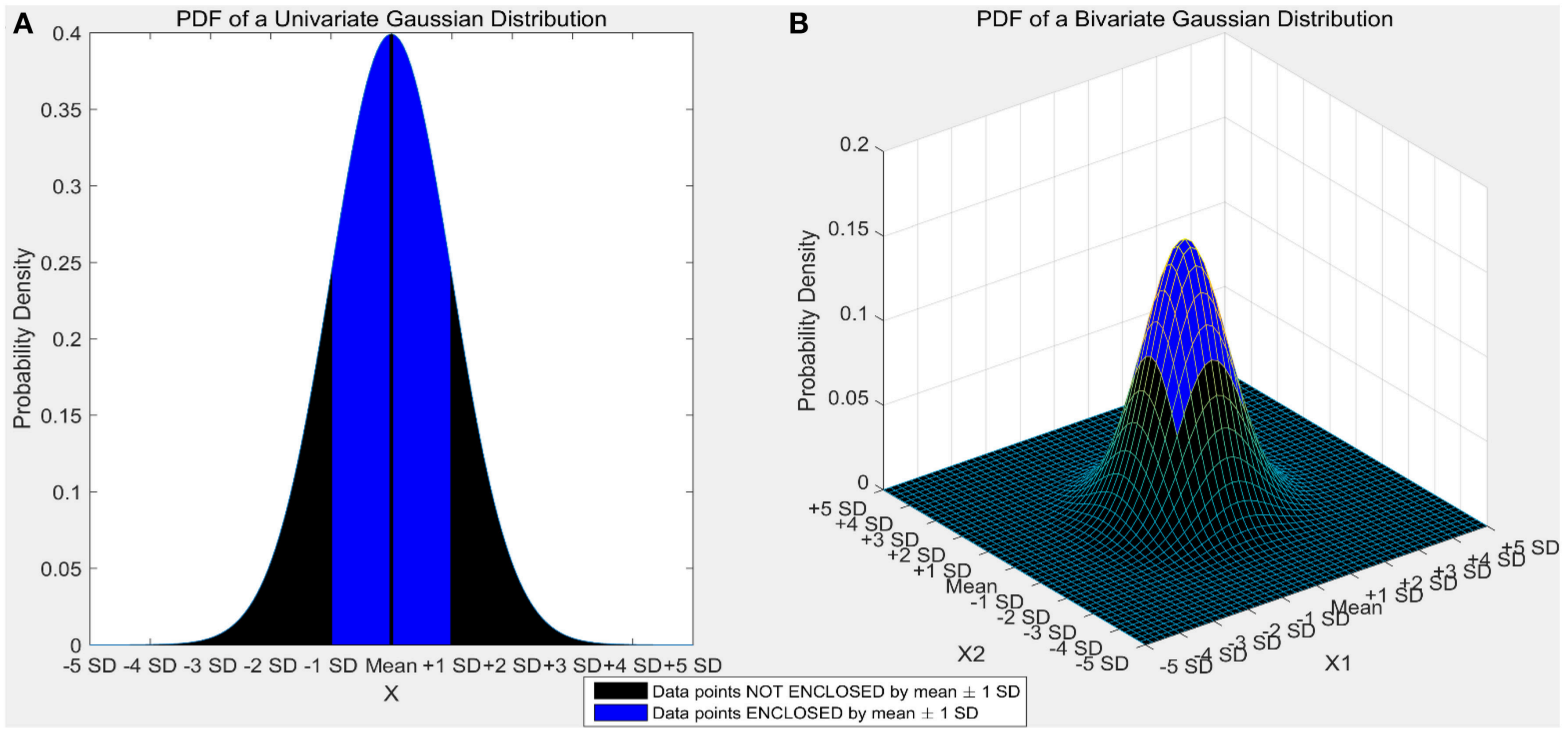
**(A)** Univariate gaussian distribution. The area shaded in red shows the data points bounded by mean ± 1 standard deviation. 68.27% of the data is enclosed in this region. **(B)** Bivariate Gaussian distribution. The area shaded in blue shows the data points bounded by mean ± 1 standard deviation. Only 39.35% of the data is enclosed in this region. As the number of dimensions increase from a univariate to a bivariate distribution, the amount of data bounded by mean ± 1 standard deviation reduces by ~42%.

**Figure 2:**
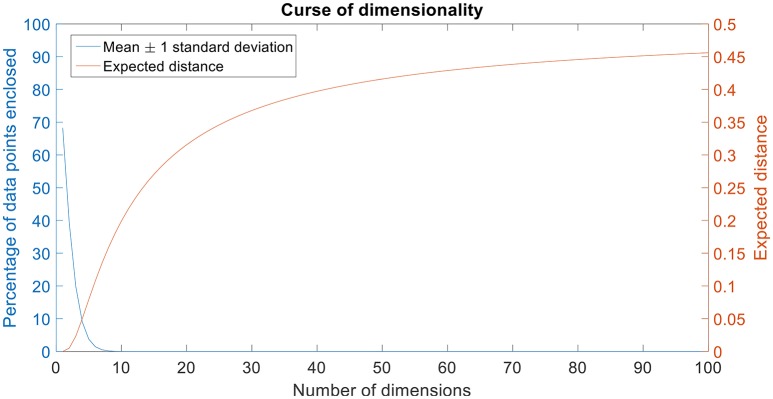
Curse of dimensionality. The plot in blue shows the percentage of data points enclosed by the mean ± 1 standard deviation for 100 dimensions. The amount of data enclosed by the mean ± 1 standard deviation region asymptotes to zero from an octa-variate (8-dimensional Gaussian distribution contains only 0.18% of the data in mean ± 1 standard deviation region) distribution. The plot in red shows the expected distance for 100 dimensions. Expected distance is defined as the average distance between the sample points and the edge of a hypercube. The expected distance asymptotes to its maximum value of 0.5 as the number of dimensions increases.

Certain machine learning algorithms fail to scale well in high dimensional feature space. These algorithms suffer from the “curse of dimensionality,” which refers to the problems that arise when analyzing and organizing high-dimensional data. Consider a univariate, independent variable “X” which follows a Gaussian distribution with mean “μ” and variance “σ” (X ~ N (μ, σ)). According to the properties of Gaussian distribution, ~68% of the data is enclosed in the region surrounded by the mean ± 1 standard deviation (Figure 1A). Consider two independent variables X_1_ and X_2_ which follow Gaussian distributions with means “μ_1_” and “μ_2_,” and variances “σ_1_” and “σ_2_” respectively (X_1_ ~ N(μ_1_, σ_1_) and X_2_ ~ N(μ_2_,σ_2_)). For a bivariate, Gaussian distribution only ~40% of the data is enclosed within the same region (Figure 1B). For a 50-dimensional multivariate normal distribution, only ~1/250,000,000th of the data lie within the mean ± 1 standard deviation region. As the number of dimensions (variables) increase, the amount of data bounded by the mean ± 1 standard deviation region decreases exponentially (Figure 2). In neural decoding, data from each electrode is treated as an individual feature. A microelectrode array usually consists of 96 electrodes thus, making the feature space 96-dimensional. In case of a 96-dimensional feature space, only an infinitesimally small proportion of data points are enclosed in the mean ± 1 standard deviation region. Results of Figure 1 were generated using a novel approach to constructing the Multi-dimensional standard deviation ellipsoid based on spectral decomposition of the sample covariance (Wang et al., 2015).

In high-dimensional space, almost every point is closer to the edge of a hypercube that encloses the points than to another sample point. For a sample of size “n,” the expected average distance between the sample points and the edge of the hypercube “D” in a “d”-dimensional feature space can be estimated using the following equation:

$$D\left(d,n\right)=\frac{1}{2}.{\left(\frac{1}{n}\right)}^{\frac{1}{d}}$$

For a two-dimensional space with 10,000 points, the average expected distance between the sample points is 0.005 and for a 100-dimensional space with the same number of points, the expected distance is 0.45. It should be noted that the maximum distance from any point to the edge is 0.5 for normalized values of dimensions (Kantardzic, 2011). The expected distance asymptotes to 0.5 when the number of dimensions approaches infinity.

It can be seen that the percentage of data points enclosed by the mean ± standard deviation region decreases as the number of dimensions increase (Figure 2). Also, the expected distance increases quadratically (and asymptotes toward its maximum value, 0.5) as the number of dimensions increase (Figure 2).

The above two examples illustrate the sparsity of finite data in high-dimensional space. In high-dimensional space, most data points act as outliers. This sparsity in data distribution deters the efficacy of certain machine learning algorithms in high-dimensions. Feature selection is one of the methods to cope with “curse of dimensionality.”

Using machine learning algorithms for multivariate, high-dimensional data is often computationally expensive. Due to the complexity of feature space and rigorous numerical computations involved in defining the hyperplane in this high-dimensional feature space, the performance of the machine learning algorithm is deterred. Feature selection is the process of selecting an *O*-dimensional subset feature space from a *P*–dimensional original feature space where “*p*” is the number of predictors.

Feature selection is usually applied to reduce information redundancy and trim the input space to better predict the responses. Some of the advantages of feature selection are:

- Facilitate data visualization and data understanding
- Reduce data measurement and storage requirements
- Reduce training and utilization times
- Simplify the learning model and aid in better understanding and interpretation by researchers
- Enhance generalization by reducing overfitting
- Defy the curse of dimensionality to improve predictor performance (Guyon and Elisseeff, 2003).

Identifying the best subset of features is a time consuming and resource intensive problem to solve. The only method to do this is through exhaustive grid search, i.e., exhaustively searching through every permutation of predictors available. Mathematically, there exists 2^p^ permutations of features that can be selected from “p” features. In case of our neural data, this results in iterating through 2^96^ (96 features for multi-unit firing rate and >96 features for single-unit firing rate based feature vector) permutations of features to identify the “best” subset.

When dealing with multivariate, time-series signals like neural signals, it is imperative to judge where the learning algorithm must focus its attention. *Filter* or *Criterion* based feature selection and *Wrapper* based feature selection are two broad categories of feature selection that are commonly applied in machine learning (Kohavi and John, 1997). Application of statistical, empirical or other “criteria” based methods such as mean, variance, student's *t*-test and correlation are some examples of criterion based feature selection. Applying criterion based feature selection requires some domain expertise in order to determine what qualifies as a useful criteria. Wrapper based feature selection iteratively uses various combinations of features as input to a machine learning algorithm and evaluates the importance of each feature based on some evaluation criteria from the prediction such as coefficient of determination (r^2^). Ideally, it is advisable to use the same machine learning algorithm as a classifier and a wrapper for feature selection. Oftentimes, it is also valuable to use a simpler, computationally efficient machine learning algorithm as a substitute wrapper. For example, SVMs are an efficient but computationally intensive solution to solve the problem of face recognition by computing key points (that act as features) on the face. Using SVM as a wrapper in this case would demand access to a lot of resources (in terms of clusters) and still be time consuming. An alternative to using SVM in this case would be using a simpler algorithm such as Logistic regression. Care should be taken to ensure both the algorithms have similar assumptions about the data such as nonlinearity or heteroscedasticity of noise.

## Methods

Approval for the animal use protocol in this study was obtained from the University of Utah Institutional Animal Care and Use Committee (IACUC). All procedures conformed to National Institute of Health (NIH) standards for animal care. The recording setup, behavioral task, data collection and preliminary data processing approaches are explained elsewhere (Baker et al., 2009). A 96 channel microelectrode array (MEA, Blackrock Microsystems) was implanted in the hand area of primary motor cortex of a male macaca mulatta. The NHP was trained to perform cued combined flexions of the thumb, index and middle finger and individual flexions and extensions of the same digits using a manipulandum. Visual cues were provided using a computer screen placed in front of the monkey. In order to start a trial, the monkey had to relax all its fingers moving all of the finger switches in the manipulandum to the open state. After a randomized wait time of 1,000–3,000 ms, a visual cue indicating which finger(s) to flex/extend appeared on the computer screen. The monkey then had 2,000 ms to react to the visual cue and depress the associated switch. Once the correct switch was pressed, the monkey had to hold the switch for 500 ms. The trial was deemed successful if the monkey pressed the correct switch and adhered to the time constraints. The behavioral task was implemented using a real-time operations systems in a custom LabVIEW (National Instruments) program.

### Neural decoding system architecture

**Figure 3:**
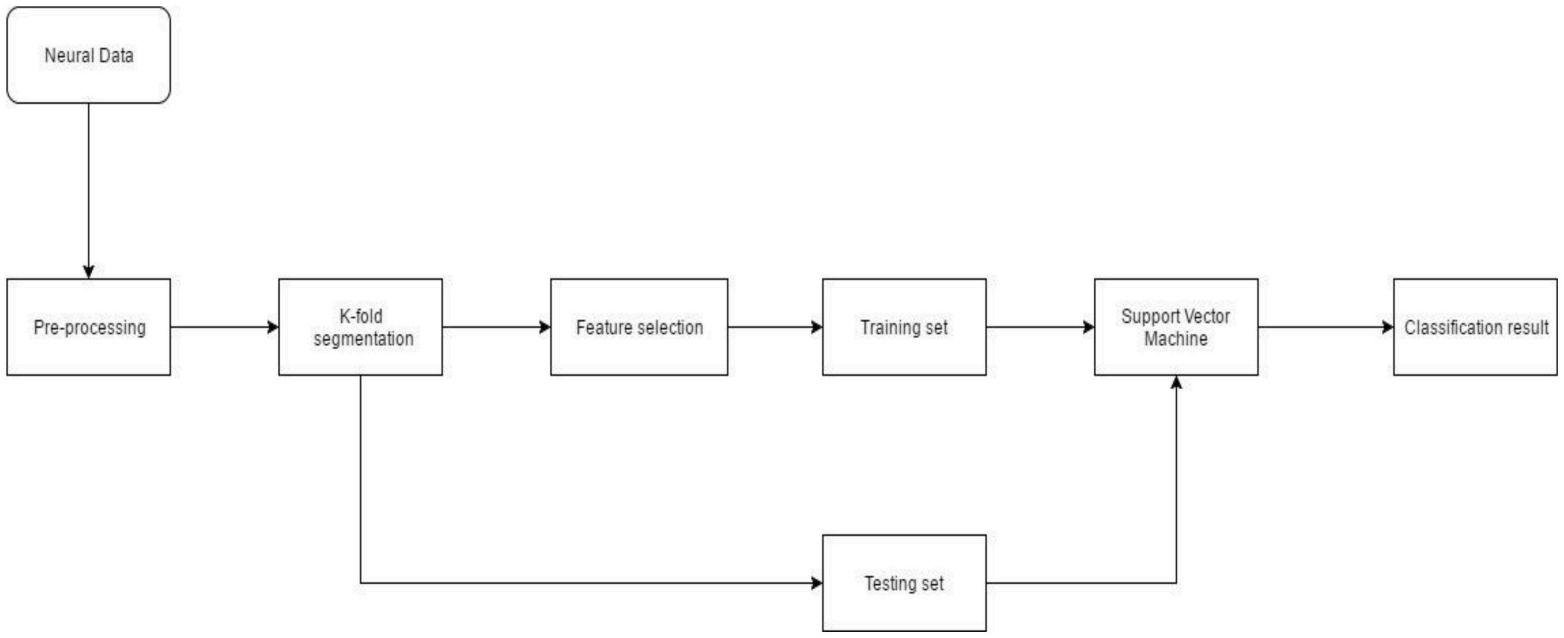
Neural decoding system architecture. After pre-processing the neural data, feature selection was performed. During K-fold segmentation, the entire data set was divided into 10-folds. 9-folds were used for training and the 10th-fold was used for validation. This process was repeated 10 times until each fold served as a validation fold. The 9-fold training data was further divided into 3-folds for parameter estimation of the SVM. Validation error and parameters of the SVM were estimated using a nested cross-validation loop.

Neural data recorded from the NHP was spike sorted. The timestamp of spike events was obtained from the offline sorter. Pre-processing also included binning/moving average windowing of the point process using a boxcar window. After applying the moving average technique, neural “firing rate” for each single or multi-unit was obtained. Neural firing rate was used as the feature vector (input) to the SVM. Neural activity corresponding to each successful finger movement trial was extracted and concatenated. The entire dataset was randomly divided into 10-folds. Each fold served as the testing set once while data from the remaining folds was used for training. Model parameters such as box constraint(C) and sigma (for the RBF kernel) were estimated using an exhaustive grid search algorithm with exponentially increasing values from 1e-5 to 1e5. Classification accuracy was calculated after predictions were made on the unseen test set. This process was repeated 20 times to reduce generalization error of the SVM (Figure 3).

### Pre-processing

The MEA is a 10 × 10 grid of 1 mm tall electrodes that are capable of recording APs in addition to LFPs (House et al., 2006). The MEA data were sampled at 30 kHz. Neural data collected using the MEA were sorted offline using an expectation-maximization based competitive mixture of *t*-distributions decomposition algorithm (Shoham et al., 2003). Data were then imported to Matlab (Mathworks) for further analysis. The time stamps of APs recorded at 30 kHz were downsampled to 600 Hz. A boxcar moving average window of 300 ms width and 33.3 ms step size was used to obtain a moving average firing rate (Davis et al., 2016). Electrodes in the motor cortex can record from more than one neuron. The features extracted from neural signals recorded from such electrodes are called “multi-unit” firing rate. However, the neural activity recorded on such electrodes can be separated using techniques such as Principal Component Analysis (PCA), Expectation-Maximization algorithm or Independent Component Analysis (Lewicki, 1998). Features extracted from such individual, isolated neurons are called “single-unit” firing rate. The moving average firing rate was downsampled in order to reduce data size. A 4th order low pass Butterworth filter with a cut-off frequency of 10 Hz was used prior to downsampling the neural firing rate to 20 Hz and the neural firing rate was obtained as a time varying vector. This process was repeated for all 96 electrodes to obtain multi-unit neural firing rate, i.e., the cumulative firing rate of all neurons recorded on a particular electrode. An average of 142.2 ± 36.3 neural units were recording from 96 electrodes during each session. Spike-sorting was performed on all neural data for each experimental recording session separately from other recording sessions.

Data from individual trials was aligned in time on switch closure times of successful trials. A movement period was defined as the duration corresponding to 450 ms prior and 1,000 ms after the switch closure. A baseline period (resting state) for a trial was defined as the duration corresponding to 2,500–1,000 ms prior to switch closure. Baseline and movement period data was obtained for all available degrees of freedom and all successful trials for each day experiments were conducted and represented a vector of time-series data.

### Feature selection

In this study, we have limited our comparisons to criteria based feature selection methods.

#### Wilcoxon signed-rank test

Wilcoxon signed-rank test is a non-parametric alternative to the student's *t*-test. This non-parametric test can be used to identify if samples from two independent yet related distributions are significantly different (Randles, 1988). In the context of selecting single or multi-unit data as input to the SVM, the difference between baseline and movement related firing rate was computed. The null hypothesis was that the data came from a continuous, symmetric distribution with a median equal to zero (i.e., no electrode recorded increased firing rates in the movement period as compared to the baseline period). Electrodes for which the null hypothesis was rejected (*p* < 0.001) with a positive median difference from baseline were kept. These electrodes were then sorted in order of increasing median difference. For the purpose of feature selection, the median difference was computed as a scalar to select features (single unit/multi-unit).

#### Relative importance

Relative importance was a feature selection technique initially developed for selecting neurons in the primary motor cortex for decoding (Kim et al., 2012). First the movement only firing rate (difference of movement and baseline firing rate) was computed. The trial averaged firing rate for each neuron for all the successful trials was calculated. Then, the inter-movement variance was computed as the difference of trial averaged firing rate and the average firing of a neuron for a particular movement. The neural recordings were then ranked in descending order of inter movement variance. For the purpose of feature selection, the inter movement variance was computed as a scalar to rank features (single/multi-unit).

#### Principal component analysis

PCA can be used as a feature transformation technique, where a transform function is applied to the data to represent it in a higher dimensional transform space. For an “*n*” dimensional possibly correlated data, PCA represents the data in a (n−1) dimensional space in linearly uncorrelated principal component coordinates (Jolliffe, 2002; Lu et al., 2007). The transformation is carried out in such a way that the first principal component contains the maximum possible variance of the data. The succeeding principal components are ordered in descending order of variance. This transformation of data according to the variance at each time point can be used to eliminate noise, but does not necessarily extract discriminative features. Neural firing rates corresponding to each movement was provided as an input to PCA. The operation of PCA can be thought of as revealing the internal structure of the data based on its variance. For a multivariate dataset that can be represented in a high-dimensional space, PCA provides a better representation in low-dimensional space from an “informative” viewpoint. This is done by considering only the first few principal components and thus, PCA serves as a dimensionality reduction method. The features extracted using PCA were ranked based on the amount of variance explained by the individual principal components.

#### Mutual information maximization

Mutual information is the mutual dependence of two random variables. Unlike correlation, mutual information is not limited to real-valued random variables and estimates how similar the joint distribution P(X|Y) is to the products of the factored marginal distribution P(X) and P(Y) (Torkkola, 2003). Entropy of a random variable C can be defined as:

$$H\left(C\right)=-\sum_{c}P\left(c\right)\text{log}(P\left(c\right))$$

The conditional entropy of two random variables C and Y can be defined as:

$$H\left(C|Y\right)=-\sum_{c}P\left(c|y\right)\log\left(P\left(c|y\right)\right)dy$$

Then, the mutual information of random variables C (neural firing rate) and Y (movement type) can be defined as the I(C;Y) = H(C) – H(C|Y) and can be represented as:

$$I\left(C|Y\right)=\sum_{c}\sum_{y}P\left(c|y\right)\text{log}\frac{P(c|y)}{P\left(c\right)*P(y)}$$

Mutual Information maximization (MIM) was implemented using the FEAST Toolbox available for MATLAB (Brown et al., 2012). For a class label Y, the mutual information score of feature C is defined as:

J(C)=I(C|Y)

This score J(C) is referred to as MIM and we rank the features in descending order of the mutual information score. Neural firing rates corresponding to movement period for each degree of freedom was used as the input to MIM algorithm.

### Support vector machine

Support vector machines (SVM) have shown promising results in upper extremity decoding tasks using various source signals such as MEG, EEG, ECoG, and EMG (Bitzer and van der Smagt, 2006; Demirer et al., 2009; Quandt et al., 2012; Wissel et al., 2013). SVM is a class of non-probabilistic, binary, linear classifier (Platt, 1999). SVMs represent the data in higher dimensional space and find the best separating hyperplane in this space. The objective of the SVM is to find a hyperplane that has the maximum distance from a point belonging to any class. Such a classifier is also called a maximum margin classifier whose generalization error is low. During training, each point in the training set is assigned a weight α. Those points with training weights α ≠ 0 are called the support vectors since, they help forming the hyperplane. In case of linearly non-separable cases, a soft margin classifier is implemented which allows for misclassified instances. Non-linear problems can be solved by using the “kernel trick” in the SVM. Kernel functions map data into a higher dimensional space where, the hyperplane is now formed. Gaussian (radial basis function) kernel was employed in our classification problem to account for non-linearity in the input-output relationship. Gaussian kernel K(x,x') for two samples x and x' defined as a feature vector in some predictor space is defined by:

$$K\;\left(x,x^{\prime }\right)\;=exp\;\left(\frac{||x-x^{\prime }|{|}^{2}}{2\sigma^{2}}\right)$$

where σ is a free parameter that defines the smoothness of the Gaussian kernel.

SVMs are inherently binary classifiers, i.e., they can distinguish between only two classes. Their functionality can be expanded to solve multiclass problems by decomposing it into multiple binary sub-problems (Hsu and Lin, 2002; Duan and Keerthi, 2005). We used a one-vs.-one multiclass implementation of the SVM to differentiate between the many available movements. For a problem of classifying “*k*” classes, we require $\frac{k\left(k-1\right)}{2}$ binary SVM classifiers for each pair of the “k” classes. The class of a test instance is predicted by taking the mode of predictions of all the one-vs.-one SVM pairs.

In addition to extracting neural activity corresponding to valid trials for all available degrees of freedom for a particular session, we included 30 random baseline periods as a “rest” phase (11th degree of freedom). During the training phase of supervised learning algorithms such as SVM, the algorithm must be provided with corresponding outputs (class labels). The class labels were created depending on the movement type. For example, thumb flexion was encoded as 1, index flexion as 2, middle finger flexion as 3 and so on.

### Performance metrics

The first step in assessing the performance of feature selection methods was to find the optimal number of features for each feature selection algorithm that best classified the different finger movements and the resting state. For this purpose, all available successful trials in a session were split into a 70% for training and the remaining 30% for testing. A 10-fold cross validation routine was performed to reduce variability in performance estimates during validation. For a given input data (multi-unit or single-unit firing rate), the features were ranked based on the results of the feature selection algorithms. The extracted features were ordered and selected in a descending order based on their ranking by each feature selection method with the best features being selected first. We iteratively incremented one feature (neural firing rate on a single electrode or from an isolated neuron) at a time and used it as an input to the classifier to identify the optimal number of features. In order to evaluate the performance of features selected at random, we also included random multi-unit and isolated unit firing rate feature to compare with the other methods.

#### Robustness to simulated failure

The performance of the brain machine interface (BMI) can be influenced by the quantity of neural information available for decode. Previous research has shown that there is a significant decrease in the signal to noise ratio of the neural signals and a steady decrease in impedance of the recording electrodes over time (Vetter et al., 2004; House et al., 2006). There can be a steady decrease in the number of electrodes that record APs, which can have a deleterious effect on BMI performance. AP recordings can also be affected due to glial scarring or electrode location changes (Frien and Eckhorn, 2000; Leopold and Logothetis, 2003; O'Leary and Hatsopoulos, 2006; Stark and Abeles, 2007; Berens et al., 2008; Jia et al., 2011). Feature selection algorithms should be robust enough to handle the sudden losses in neural information over time. In order to test the endurance of the feature selection algorithms, we randomly dropped 10's of percent of the available neural firing rate and tested its performance. The random removal procedure was repeated 20 times to reduce generalization bias.

#### Longevity of neural decodes

BMI are devices which will be used over an extended period of time. In order to be useful the neuroprosthetic device must be capable of accurate performance over this extended period of time. We present here the chronic decoding results of 47 sessions collected over 142 days. Spike-sorting was performed individually on each of the 47 sessions. For a given session the optimal number of features was computed. Decoding accuracy for a feature selection algorithm on a particular day was then calculated using the cross validated optimal features.

## Results

### Quality of neural recordings

**Figure 4:**
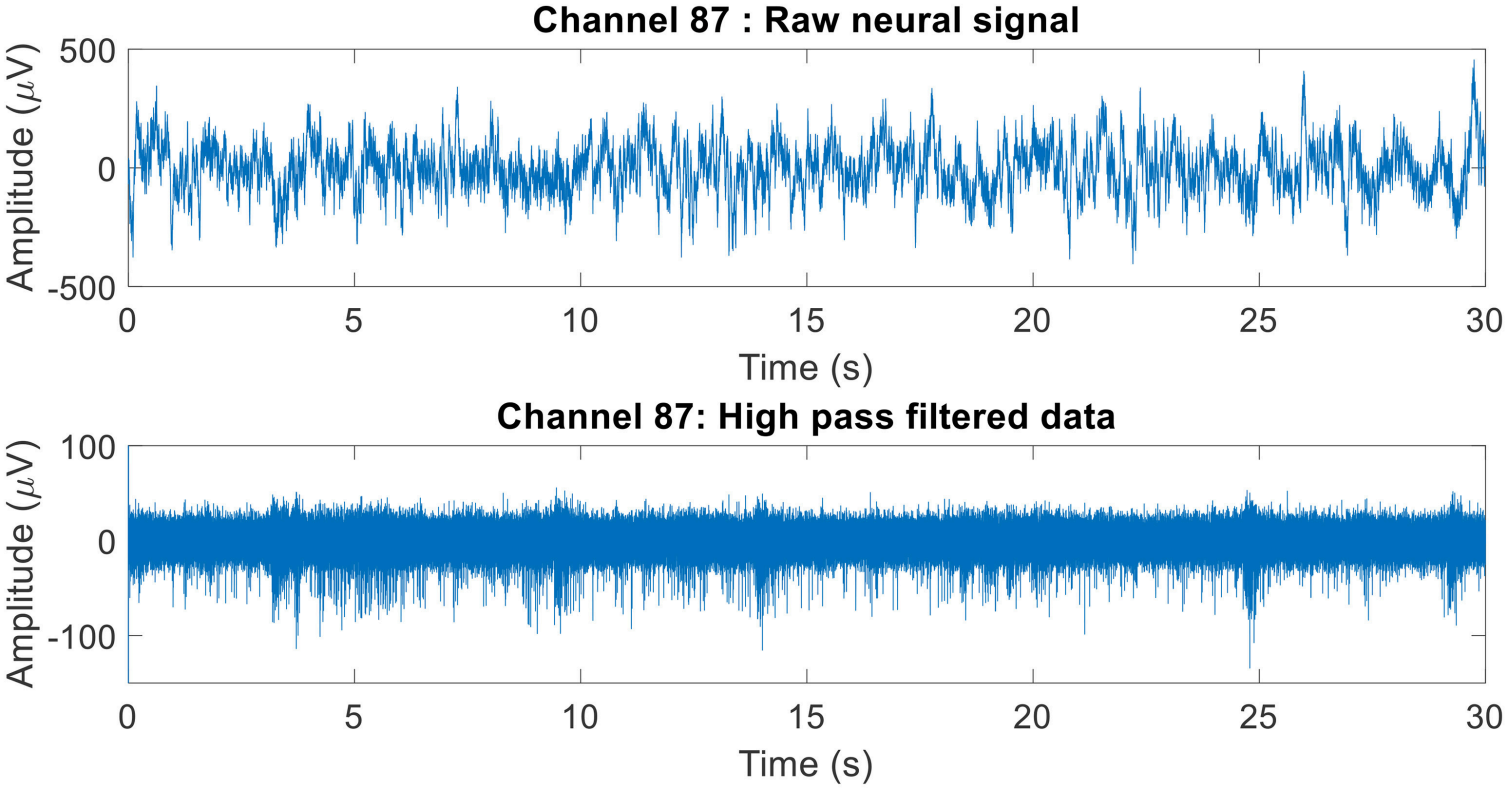
Raw neural recording on channel 87. The 30 s recording consisted of multiple different trials. The raw neural recording on each was filtered using a high-pass Butterworth of cut-off frequency 250 Hz (channel 87 was chosen at random). The MATLAB function “*filtfilt*” was used to filter the neural signal to ensure zero-phase distortion.

**Figure 5:**
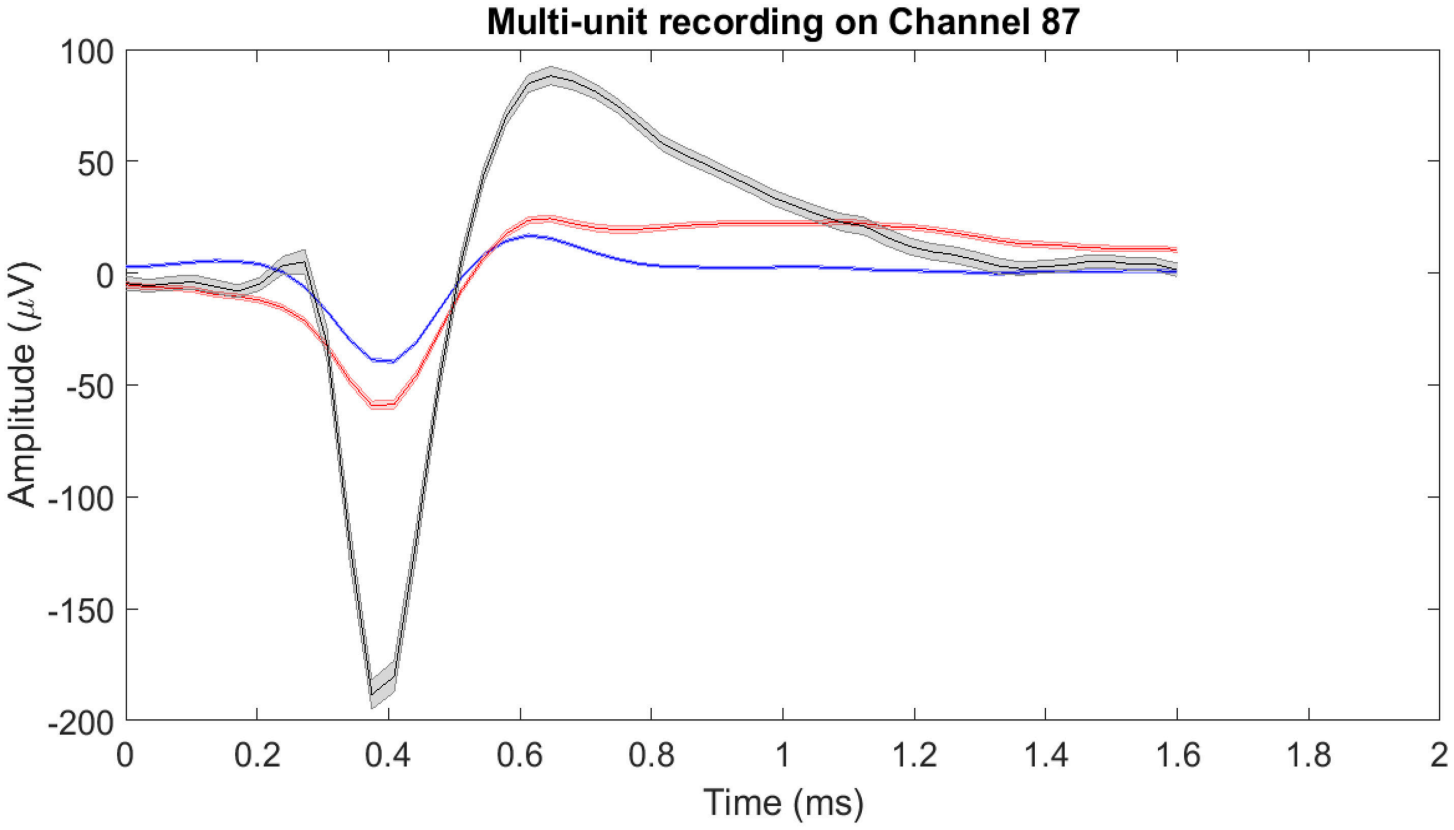
Mean of isolated single-units neural activity on Channel 87. Channel 87 contained three individual single-units post-spike-sorting. Since there was more than one single-unit recorded on channel 87, the single-unit and multi-unit firing rates were different. For single-unit firing rate, each single-unit was treated as an individual source of information. Whereas for multi-unit firing rate, the single-units were treated as one and the firing rate was computed. The shaded region around the mean action potential corresponds to the standard error of the action potential waveforms.

The raw neural data was high-pass filtered using a Butterworth filter with a cut-off frequency of 250 Hz (Figure 4). To demonstrate the quality of neural recordings used in this analysis, we extracted a 30 s neural recording on a randomly selected channel (channel 87). PCA followed by k-means clustering was performed to separate the isolated units and noise. In Channel 87, there were 3 isolated single-units (Figure 5). Single-unit firing rate was obtained by treating the isolated neural units as individual sources of information. Therefore, we obtained three single-unit firing rates for channel 87 by treating single-units 2, 3, and 4 as individual sources of information. Multi-unit firing rate was obtained by treating the individual single-units as one source of information. Therefore, we accrued the neural activity of the single-units and obtained one multi-unit firing rate for channel 87. To summarize, the number of multi-unit features is equal to the number of active electrodes (irrespective of the number of isolated units it was recording). Whereas, the number of single-unit features is equal to the number of isolated units. Spike sorting was performed individually on all data from each session.

**Figure 6:**
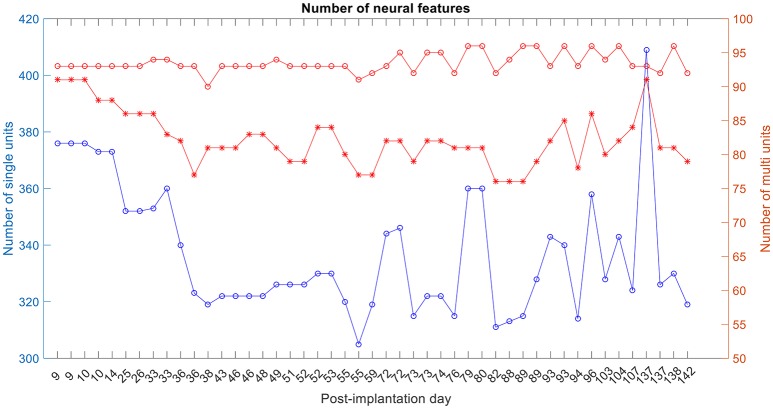
Number of single-unit features, multi-unit features and number of active electrodes with any neural activity. The blue plot corresponds to the number of isolated single units, red-asterisk plot corresponds to the number of active electrodes with multi-unit activity and the red-circle plot corresponds to the number of active electrodes with any neural recording (single and/or multi-units).

The number of single and multi-unit features was calculated (Figure 6). For the 1st session on post-implantation day 9 there were 92 electrodes (red-circle plot; out of a possible 96) with any neural activity (single and/or multi-unit recordings), 89 electrodes (red-asterisks; out of 92) had multi-unit recordings, i.e., more than one isolated single unit, and 378 isolated-single units (on the 92 electrodes).

### Selecting optimal number of features

**Figure 7:**
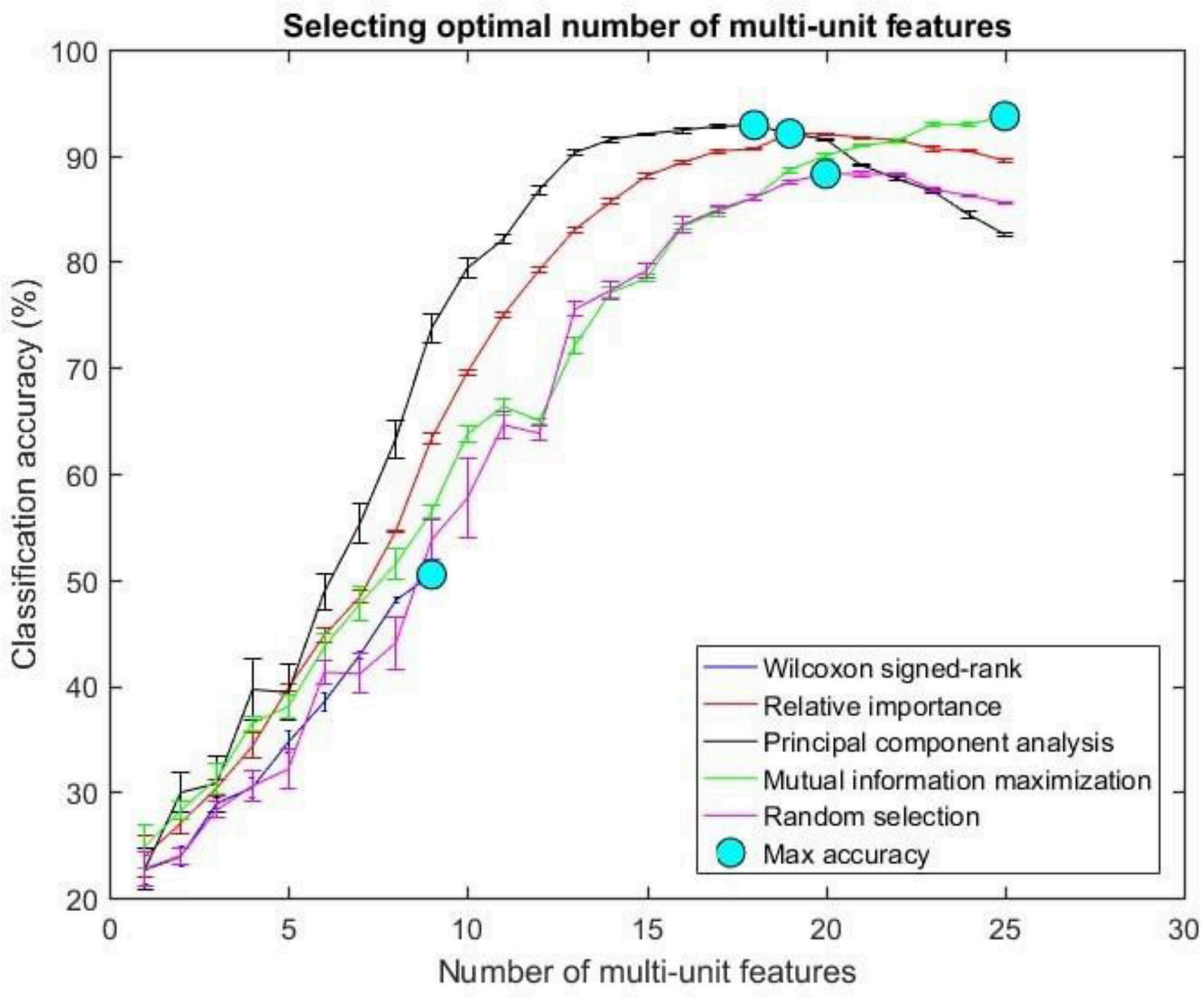
Selecting optimal number of multi-units. The plot above shows the cross validated accuracy of feature selection algorithms for increasing number of multi-unit features. The solid circle (cyan) in each graph shows the maximum cross-validated accuracy for a feature selection algorithm. The number of single or multi-unit features corresponding to this accuracy was chosen as the optimal number of features. The points and error bars correspond to the mean and standard error of maximum cross-validated accuracy respectively.

**Figure 8:**
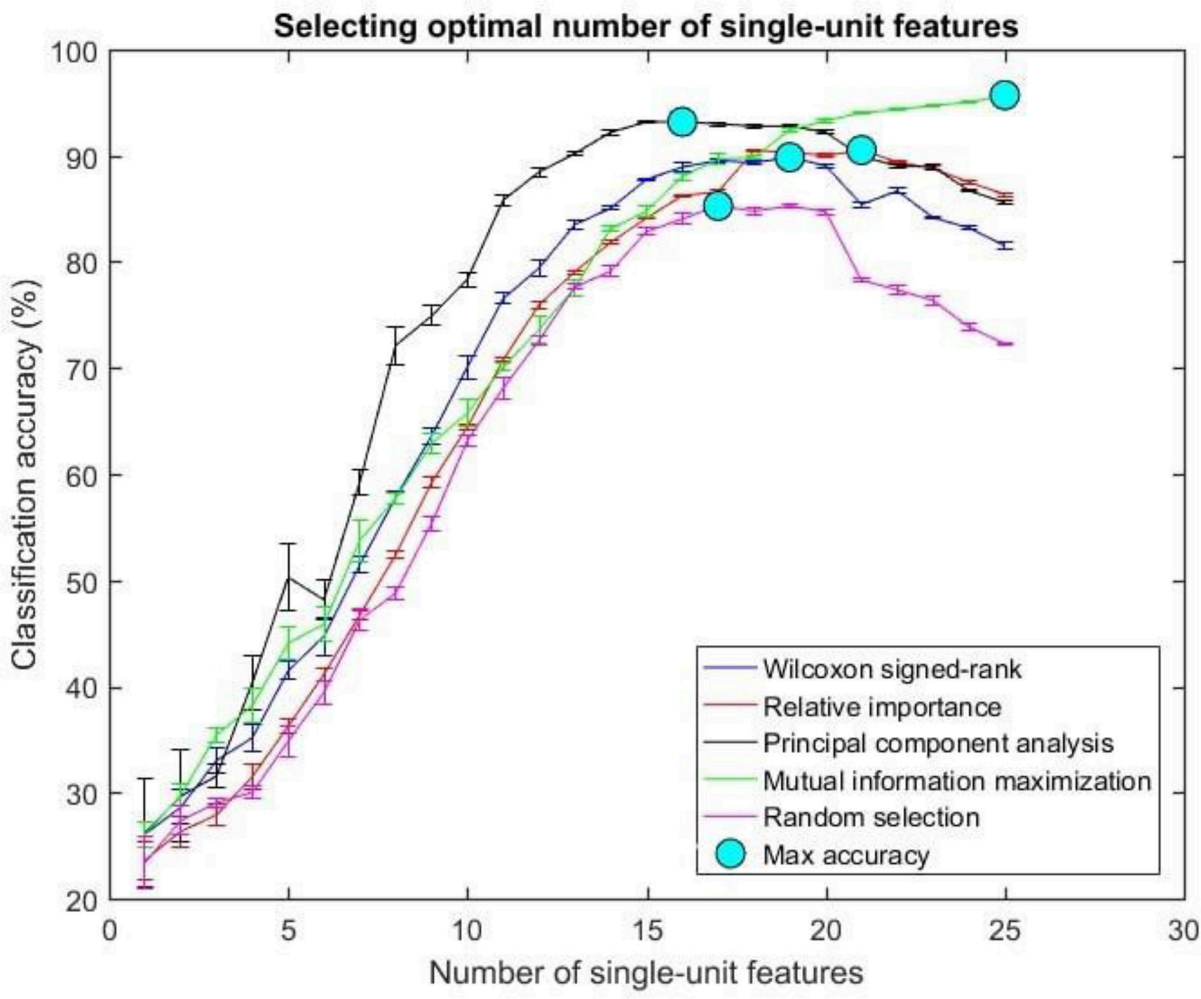
Selecting optimal number of single-units. The plot above shows the cross validated accuracy of feature selection algorithms for increasing number of single-unit features. The solid circle (cyan) in each graph shows the maximum cross-validated accuracy for a feature selection algorithm. The number of single or multi-unit features corresponding to this accuracy was chosen as the optimal number of features. The points and error bars correspond to the mean and standard error of maximum cross-validated accuracy respectively.

The optimal number of features for various feature selection algorithms on post-implantation 36 was differing (Table 1). The classification accuracy for incremental values of number of features was plotted (Figures 7, 8).

**Table 1:** Feature selection algorithms and their respective optimal number of features on post-implantation day 36.

	**Wilcoxon signed-rank test**	**Relative importance**	**PCA**	**MIM**	**Random features**
Multi-unit	9 (50.48%)	19 (92.07%)	18 (92.88%)	25 (93.71%)	20 (88.28%)
Single-unit	19 (89.84%)	21 (90.53%)	16 (93.19%)	25 (95.71%)	17 (85.27%)

The values in Table 1 correspond to the optimal number of features (values outside the parentheses) and maximum cross-validated accuracy (values within the parentheses). With an exception of Wilcoxon signed-rank test, the other feature selection algorithms did not show significant changes (two sample *t*-test, *p* < 0.05) from using multi-unit and single-unit firing rate both in terms of number of optimal features and classification accuracy (less than ± 3% difference in classification accuracy and ± 1 feature). In case of Wilcoxon signed-rank test, the number of optimal features increased from 9 features for multi-unit firing rate to 19 feature for single-unit firing rate. The classification accuracy improved from 51.12 ± 0.65% for multi-unit firing rate to 88.12 ± 0.61% for single-unit firing rate (Figures 7, 8). The number of optimal features for multi-unit features using Wilcoxon signed-rank test stops at 9 features because this feature selection methods returned only 9 multi-unit features as having a significant difference between the movement and baseline period.

**Figure 9:**
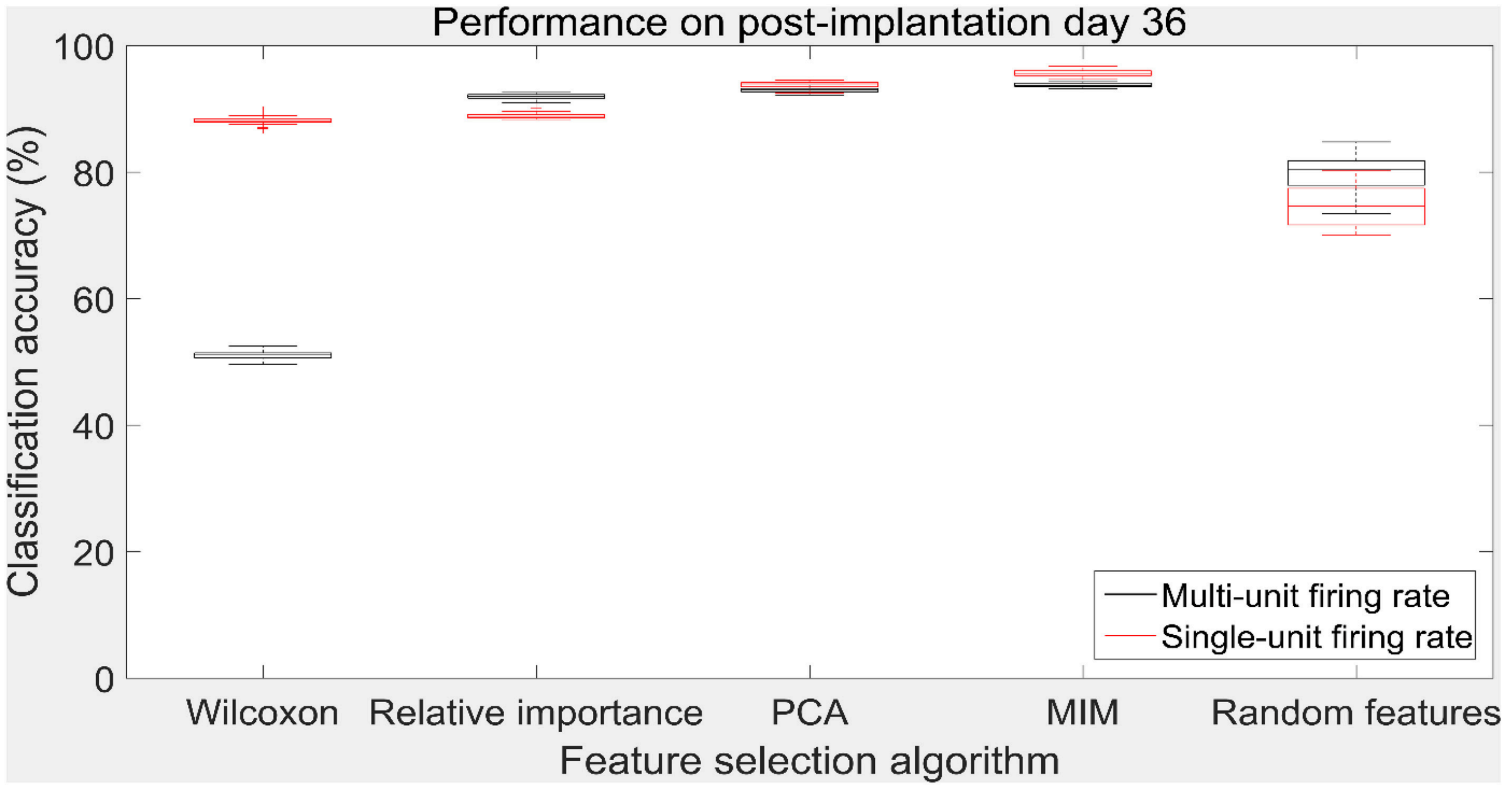
Accuracy of neural decode on post-implantation day 36. Classification accuracy of feature selection algorithms on the test set using cross validated optimal number of features. The plots in black and red correspond to classification accuracy obtained using multi-unit firing rate and single-unit firing rate respectively. Level of chance was 10% (10 degrees of freedom). The central box represents the central 50% of the data with the top and bottom sides of the central box representing the 75% quantile and 25% quantile respectively. The central line in the central box represents the median of the central 50% of the data. The vertical lines extending above and below the central box represent the remaining data that are not regarded as outliers.

The performance of the various feature selection methods was analyzed on a randomly selected session (post-implantation day 36). On post-implantation day 36, MIM performed significantly better than the other algorithms and random selection (two sample *t*-test, *p* < 0.05, α – values calculated using Bonferroni correction to account for multiple comparisons correction). There was no significant difference in the performance of single-unit and multi-unit features selected using PCA and MIM (Two sample *t*-test, *p* < 0.05). Whereas, single-unit features selected using Wilcoxon signed-rank test and Relative Importance performed significantly better than the multi-unit features selected using the respective algorithms (Figure 9).

### Robustness to simulated failure

**Figure 10:**
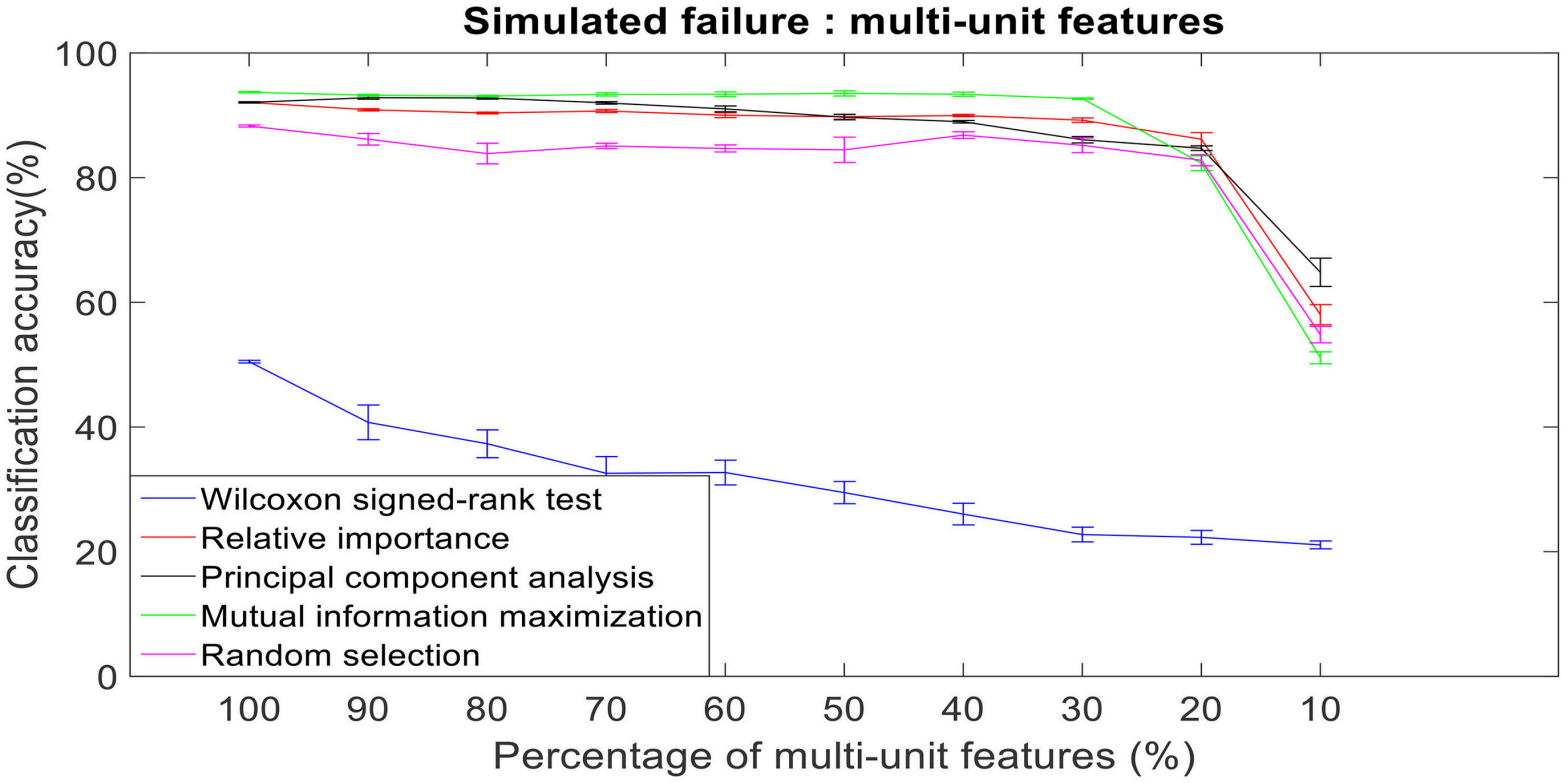
Robustness to simulated failure of multi-unit features. The plot above shows the cross-validated classification accuracy at various failure levels. Error bars indicate standard error of the cross validation folds.

**Figure 11:**
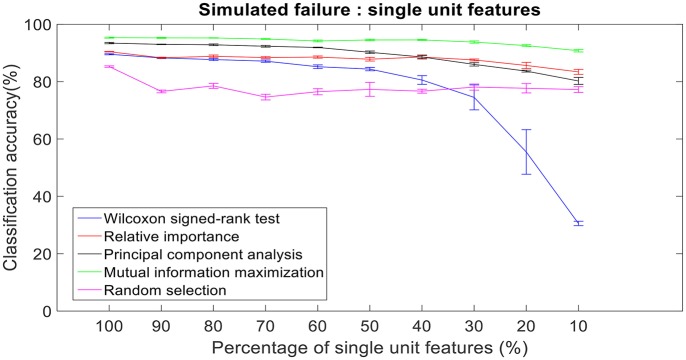
Robustness to simulated failure of units. The plot above shows the cross-validated classification accuracy at various failure levels. Mutual information maximization based feature selection had a classification accuracy of 90.79% with just 10% of the neural units as feature vector. Error bars indicate the standard error of classification accuracy.

There was a total of 96 multi-unit features and 350 single-unit features from neural data recorded from post-implantation day 36 available for this analysis (corresponding to 100% of features). Therefore, for the 10% case there was 9 multi-unit features and 35 single-unit features. There is a general trend of decrease in the performance of feature selection algorithms when we decrease the number of features from 100 to 10%. While using multi-unit firing rate, the performance of PCA was best at 64.82 ± 2.27% for 10% of multi-unit features, whereas the performance of Wilcoxon signed-rank test was 21.08 ± 0.63%. When we used single-unit firing rate as the feature vector, the robustness to simulated failure was higher for all feature selection algorithms when compared to their respective multi-unit firing rate. In case of Wilcoxon signed-rank test there was a ~10% increase in classification accuracy while there was a ~40% increase in classification accuracy for MIM based feature selection. The performance of MIM feature selection for single-unit firing rate stayed above 90% classification accuracy even while using only 10% of the available units. MIM based single-unit features performed significantly better than all of the other algorithms for all levels of simulated failure (100–10%) (Kruskal-Wallis test, *p* < 0.05) (Figures 10, 11).

### Longevity of neural decodes

**Figure 12:**
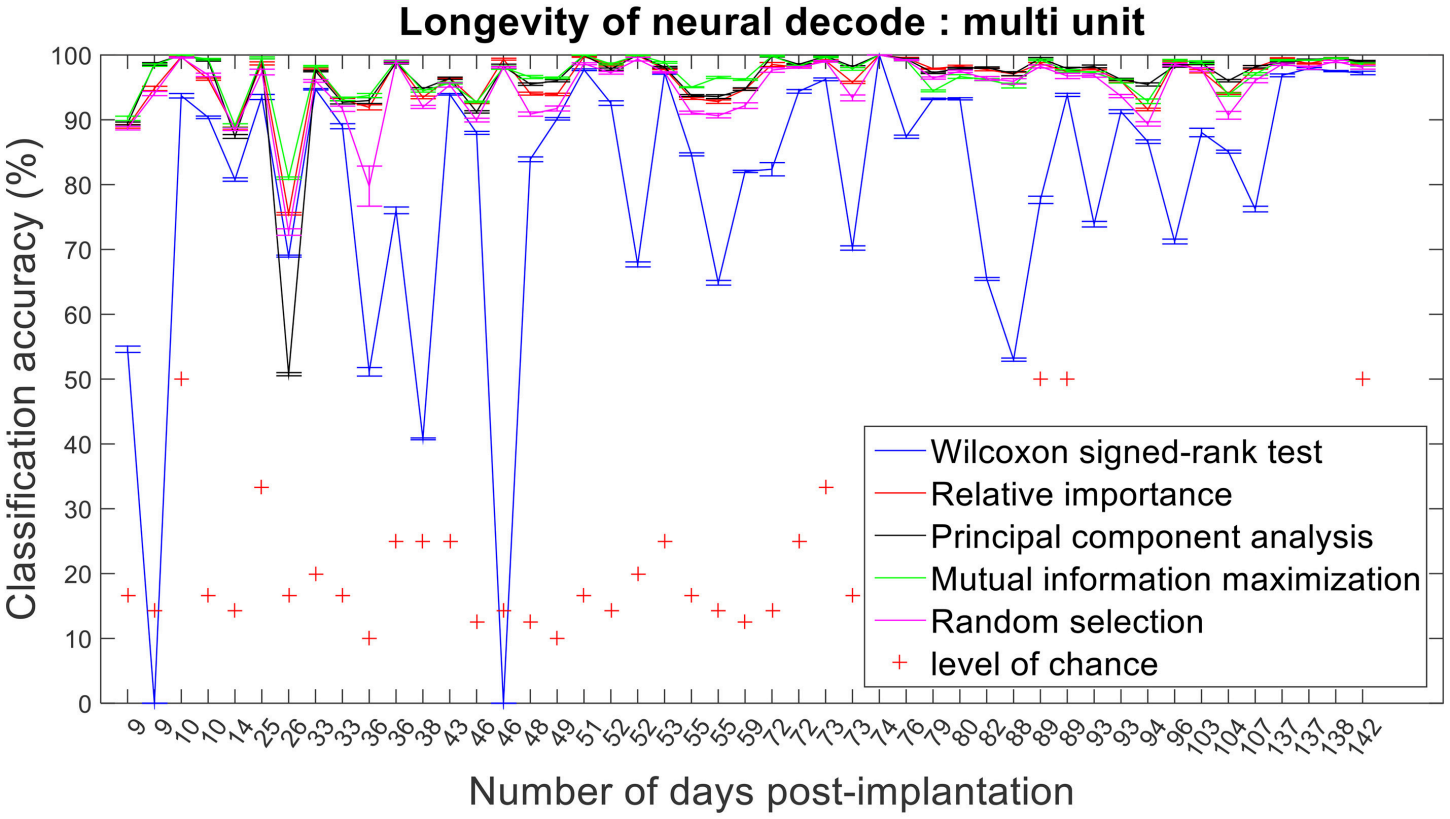
Longevity of neural decodes using multi-unit firing rate. High levels of accuracy over an extended period of time is imperative for a fully functional neuroprosthesis. On certain days, two sessions of recordings were conducted. Repeated x-axis indices (Number of days post-implantation) in the above figure correspond to different sessions conducted on the same day.

**Figure 13:**
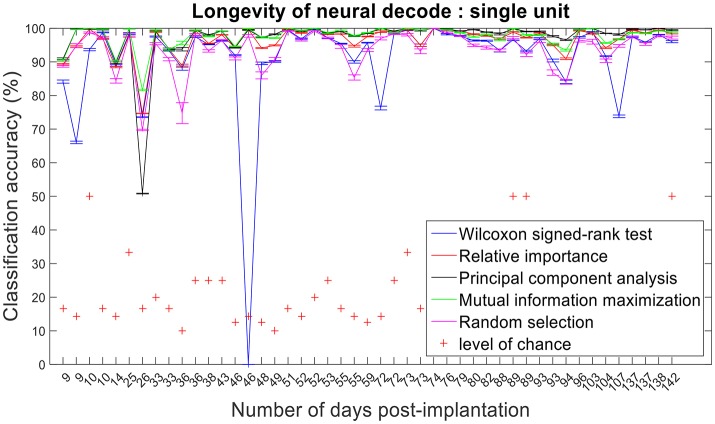
Longevity of neural decodes using single-unit firing rate. The optimal number of features for each feature selection technique was identified using an iterative cross validation scheme. For a given day, cross validation and performance evaluation were computed as described in section Performance Metrics.

The improvement in classification accuracy from multi-unit to single-unit firing rate requires an AP isolating pre-processing procedure. Relative importance, PCA and MIM had comparable accuracies across 47 sessions and performed better than randomly selected features (Figures 12, 13).

Assessing the chronic decoding capability of various feature selection methods, MIM produced the best results for both single-unit and multi-unit based firing rate (Table 2). The decoding accuracies of MIM based feature selection was compared to the other methods used in this analysis (two sample *t*-test, *p* < 0.05, α – values calculated using Bonferroni correction to account for multiple comparisons correction). In general, single-unit firing rate feature vector yielded slightly better (~3–4% on average) performance compared to multi-unit firing rate feature vector for all feature selection methods except Wilcoxon signed-rank test. The chronic decoding results also validate the viability of using a neuroprosthetic device with high classification accuracies (>90% classification accuracy on average).

**Table 2:** Longevity of neural decoding.

	**Wilcoxon signed-rank test**	**Relative importance**	**Principal component analysis**	**Random selection**
Multi-unit	46	21	13	45
Single-unit	45	32	13	45

*The values in this table correspond to the number of sessions during which MIM yielded higher decoding accuracies compared to the other methods*.

Isolating the APs from individual neurons is routinely performed on neural recordings from microelectrodes. We have shown that by applying feature selection techniques to single-unit and multi-unit firing rates, we can get comparable performance over a chronic level. However, utilizing single-unit firing rates demonstrated better performance than multi-unit firing rates when the number of active electrodes decreased.

## Discussion

Feature selection is an efficient method to cope with the “curse of dimensionality.” As explained in the previous sections, performing feature selection increases the amount of data that is bounded by the mean ± 1 standard deviation region. Reducing the dimensionality of neural data from a few hundred features to an average of 20 features, increases the amount of data bounded by the mean ± 1 standard deviation region exponentially. Therefore, the sparsity of data points in the feature space is reduced. In addition to reducing the sparsity, feature selection algorithms also inundate the feature space with more relevant information based on some criteria (Guyon and Elisseeff, 2003). In a way, feature selection can be thought of as a procedure to “prune” the feature space with only “informative” features. All the feature selection algorithms consistently performed better than the randomly selected features. This significant improvement in performance adds ~10% accuracy in case of both multi-unit and single-unit features compared to randomly selected features. Ideally in real world applications, we would expect the prosthesis to work with 100% accuracy for all different types of movement. To increase user compliance and ease of use, feature selection algorithms must yield accuracies as close to 100% as possible. Misclassifications in prediction can impede or in the worst-case cause physical damage to the user and/or people around them. Misclassifications in real time prediction can lead to undamaging mishaps that might still be critical in accomplishing tasks such as slips while holding a cup of coffee or other objects that might steer the user away from efficiently using the prosthesis for activities of daily living. We also speculate that with increasing misclassifications, user acceptance and performance might deteriorate non-linearly.

Feature selection algorithms operate in various mechanisms and perform significantly better than level of chance and randomly selected features. While Wilcoxon signed-rank test, Relative Importance and MIM retain the innate properties of the feature space (in terms of retaining it in time domain), PCA transforms the features to uncorrelated, orthogonally located principal component axes. It is interesting to note that for many sessions, PCA has comparable performance as MIM. Exploiting this property of PCA and the noise reduction it provides innately, it will be interesting to program algorithms that do not require re-training for each session. This would be a significant improvement in terms of user experience since training time is usually of the order of 20–30 min (performing the training trials and parameter selection for the feature selection and machine learning algorithm) which might be monotonous and tiresome.

Global models, i.e., models that are trained on multiple subjects and then tested on data from an unseen test subject are used for categorizing subjects into groups, e.g., diagnosis. For such an application, a subject-wise cross validation approach is preferred. Subject specific models, i.e., models that are trained on multiple segments of past data and then tested on unseen current data in a single subject are used for estimation of the current state of a given subject, e.g., prognosis. The appropriate approach is needed to approximate the use-case in machine learning (Saeb et al., 2017). We are developing a model that is unique to each subject, therefore, the appropriate method of cross validation is by partitioning the training and testing sets sequentially based on time from an individual subject rather than across multiple subjects. It is also not scientifically accurate to group data from different subjects as the placement of the electrode grids relative to the anatomy of the motor cortex will vary from subject to subject, resulting in a unique spatial sampling of the neurological data from each subject. Using a subject specific approach an intracortical prosthesis allowing people with paralysis to communicate using a virtual keyboard been demonstrated (Pandarinath et al., 2017). We believe that developing subject specific models is the appropriate method for developing BCIs.

One of the limitations of developing subject specific models for clinical applications is the lack of generalization across subjects. The data used in this study was recorded from the primary motor cortex of a healthy NHP. The primary motor cortex is a relatively well-understood part of the brain where the firing of APs is correlated and causally related to movement. The somatotopic and cytoarchitectonic structure of the primary motor cortex is conserved across primates. Therefore, it is a fair assumption that the primary motor cortex of this animal is a standard representation of the primary motor cortex of primates. Although it is impossible to predict the global performance of an algorithmic approach *a priori*, given the conserved structure of the primary motor cortex, we believe that general trends presented in our analysis will still be transferable across subjects and to similar neuroprosthetic applications.

In this study, we have tested the feature selection algorithms based on scenarios encountered with real-world neural data. Loss of active single and multi-units over a long duration of time has been observed and reported in various studies. In order to make a neuroprosthesis commercially and practically viable, the algorithm must be robust to handle reduction of available features. We have reported the performance of feature selection algorithms when subjected to a reduced subset of features. We achieved accuracies several folds above level of chance with only 10% of the single-unit features using MIM based feature selection. One of the main reasons for the loss of active single and multi-units is due to physiological interactions at the tissue-electrode interface. With technology available at our disposal today, the only way to cope with such physiological interactions might be to replace the micro-electrode array itself. Feature selection algorithms help sustain the performance of the neural decode and maximize the classification accuracy when encountering such intractable conditions.

During some chronic microelectrode array implantations, several studies have reported losing neural information (Frien and Eckhorn, 2000; Leopold and Logothetis, 2003; O'Leary and Hatsopoulos, 2006; Stark and Abeles, 2007; Berens et al., 2008; Jia et al., 2011). In this paper, we compared the performance of multi-units and single-units neural features to simulated failure. Single-unit features were more robust to simulated failure than multi-unit features. We speculate that multi-unit features performed poorer than single-unit features due to aggregation of neural information from the underlying single-units. The amplitude and frequency of firing of the underlying single-units play a significant role in helping decode motor movements. Single-units with unique information could be masked by other single units with higher frequency of firing, therefore, increasing the chance of redundant information when viewing neural information as multi-units. Using single-units might help provide information that results in higher separability of the classes, especially when the number of neural units decreases (like in a simulated failure model).

Neural decoding algorithms must also be reliable over a long duration of time. In this paper, we also present results of various feature selection algorithms over 47 sessions of neural decode. Across all the sessions, single-unit and multi-unit features had comparable performances for multiple types of movements. According to our results, for 60% of the sessions there was a significant difference between the performance of single and multi-unit features. Although there is an average ~5% increase in performance when using single-unit features, it comes with a trade-off of expensive computation. This computational latency can also manifest in the form of execution delays of the neuroprosthesis while performing a task which might directly affect user performance. We speculate that MIM performs better across all three performance metrics as it maximizes the class conditional entropies of features in the predictor space. Future analysis will investigate the stability of neural decodes. Stability of neural decodes refers to the performance of a trained model over time without updating the model. The stability of neural decoding models will impact how often a user will need to retrain the classifier model.

## Ethics statement

This study was carried out in accordance with the recommendations of IACUC. The protocol was approved by the University of Utah.

## Author contributions

SP and BG designed the methodology, performed the analysis and wrote the manuscript. JB and BG designed and performed the experiments and collected data used in this analysis. BG is the senior author.

### Conflict of interest statement

The authors declare that the research was conducted in the absence of any commercial or financial relationships that could be construed as a potential conflict of interest.
