# Corrigendum: Feature Selection Methods for Robust Decoding of Finger Movements in a Non-human Primate

**DOI:** 10.3389/fnins.2018.00420

**Published:** 2018-06-19

**Authors:** Subash Padmanaban, Justin Baker, Bradley Greger

**Affiliations:** ^1^School of Biological and Health Systems Engineering, Arizona State University, Tempe, AZ, United States; ^2^Viscus Biologics, Cleveland, OH, United States

**Keywords:** feature selection, neural decoding, principal component analysis, non-human primate, support vector machine

In the original article, we neglected to include the funder Congressionally Directed Medical Research Programs (CDMRP), W81XWH-14-1-0456 to BG. The authors apologize for this error and state that this does not change the scientific conclusions of the article in any way.

The original article has been updated.

## Conflict of interest statement

The authors declare that the research was conducted in the absence of any commercial or financial relationships that could be construed as a potential conflict of interest.

